# A survey of respiratory physicians on investigating and managing tuberculous pleuritis

**DOI:** 10.1038/s41598-026-57407-3

**Published:** 2026-06-10

**Authors:** Ken Ka Pang Chan, Hongtao Niu, Chin Chung Shu, Ying-Chun Chien, Larry Ellee Nyanti, Nai-Chien Huan, Cary Amiel G. Villanueva, Aparna Chatterjee, Debraj Jash, Jack Kit Chung Ng, Kwun Cheung Ling, Roland Leung, David Shu Cheong Hui, Pyng Lee

**Affiliations:** 1 Department of Medicine & Therapeutics, Faculty of Medicine, Chinese University of Hong Kong, Hong Kong, China; 2 Li Ka Shing Institute of Health Sciences, Faculty of Medicine, Chinese University of Hong Kong, Hong Kong, China; 3 State Key Laboratory of Respiratory Health and Multimorbidity, National Clinical Research Center for Respiratory Diseases, National Center for Respiratory Medicine, Beijing, China; 4 Institute of Respiratory Medicine, Chinese Academy of Medical Sciences, Beijing, China; 5 National Office for Chronic Respiratory Disease Prevention and Control, Beijing, China; 6 Department of Pulmonary and Critical Care Medicine, Center of Respiratory Medicine, China-Japan Friendship Hospital, Beijing, China; 7 Department of Internal Medicine, National Taiwan University Hospital, Taipei, Taiwan; 8 Medical Department, Faculty of Medicine and Health Sciences, Universiti Malaysia Sabah, Kota Kinabalu, Malaysia; 9 Department of Respiratory Medicine, Queen Elizabeth Hospital, Kota Kinabalu, Sabah Malaysia; 10 Division of Pulmonary Medicine, Department of Medicine, Philippine General Hospital, University of the Philippines, Manila, Philippines; 11 Manipal Broadway Hospital, Kolkata, West Bengal India; 12 Department of Medicine, National University of Singapore, Singapore, Singapore

**Keywords:** Adenosine deaminase, *Mycobacterium tuberculosis*, Pleural effusion, Survey, Tuberculous pleuritis, Diseases, Health care, Medical research, Microbiology

## Abstract

Diagnosing tuberculous pleuritis (TBP) is challenging. No consensus currently guides its workup and management in tuberculosis (TB)-endemic regions. An online survey was conducted to assess real-world practices of respiratory physicians when approaching new-onset unilateral pleural effusion and the likelihood of TBP being considered in the diagnostic workup. Responses from 414 respiratory physicians across 15 countries/regions were analyzed, with 98.8% from intermediate-to-high TB burden areas. TBP was frequently considered as a differential diagnosis for new-onset unilateral pleural effusion by 80.4% of respondents. Heterogeneity was observed in the diagnostic and therapeutic approaches among respondents. Initial investigations, including pleural fluid *Mycobacterium tuberculosis* (MTB) culture, adenosine deaminase and pleural biopsy for histology, were performed variably. Selection of TBP-specific diagnostic tests was correlated with regional TB burden and initial consideration of TBP. Varied perceptions of diagnostic test accuracy were also evident among respondents. A definitive TBP diagnosis, supported by histological or microbiological proof, was required by 60.9% of respondents before anti-TB treatment was initiated. 77.8% of respondents had experienced a revision of the diagnosis of probable TBP cases following empirical anti-TB treatment failure. These findings highlight the need to strengthen the diagnosis and management of TBP in surveyed regions.

**Supplementary Information:**

The online version contains supplementary material available at 10.1038/s41598-026-57407-3.

## Introduction

According to the 2025 Global TB Report by the World Health Organization (WHO)^1^, tuberculosis (TB) is one of the leading causes of death among infectious diseases worldwide. Approximately one-fifth of TB patients present with extrapulmonary TB^2,3^, of which TB pleuritis (TBP) is one of the most common forms, both within and beyond Asia^4–8^.

TBP diagnosis is often delayed because *Mycobacterium tuberculosis* (MTB) involvement in the pleural space is paucibacillary^3,9^. Pleural fluid MTB culture is positive in only 30–50% of cases^3,9^. Although pleural tissue histology can expedite diagnosis, closed pleural biopsy is limited by risks of bleeding and lung injury, needle inaccessibility, and the lack of expertise. TBP is suspected based on compatible clinical presentations and demographics, lymphocytic exudate on pleural fluid analysis, and exclusion of malignancy. These findings typically prompt physicians to start empirical anti-TB treatment^3,9^. Molecular diagnostics with polymerase chain reaction (PCR) assays, such as Xpert MTB/RIF Ultra, offer rapid detection of MTB and rifampicin resistance directly from pleural fluid. Pooled sensitivity is 68% and specificity exceeds 95% in TBP using culture-based reference standards^10^. However, performance is suboptimal in culture-negative pleural fluid^10^, and widespread use is limited by cost and infrastructure requirements in resource-constrained settings.

Many high TB burden countries are located in the Asia-Pacific region. To address these challenges, we conducted a survey to explore the real-world approach of respiratory physicians on the evaluation of a unilateral pleural effusion, their likelihood of considering TBP, and practice variability between regions with high and low-to-intermediate TB prevalence.

## Materials and methods

This multinational cross-sectional survey targeted respiratory physicians of all experience levels. The questionnaire was designed to capture heterogeneity in clinical practice approaches to TBP. It collected data on practice experience, consideration of TBP when investigating new-onset unilateral pleural effusion, availability and utilization of TBP diagnostic tools, perceived diagnostic accuracy of TBP tests, and factors influencing the initiation of empirical anti-TB treatment for probable TBP. The questionnaire was developed with input from respiratory physicians specializing in pleural medicine from mainland China, Hong Kong, India, Malaysia, Philippines, Singapore, and Taiwan. Its applicability and readability in English (Supplementary Material), Traditional Chinese, and Simplified Chinese were confirmed after a pilot trial. The online questionnaires were constructed using the Qualtrics XM platform (Qualtrics, Provo, UT, USA), a cloud-based survey tool (https://cuhk.qualtrics.com/).

Questionnaires were disseminated via email and social media, including WhatsApp, Viber, Facebook, and LinkedIn, to respiratory physicians in regions with intermediate to high TB burden (e.g., China, Hong Kong, India, Indonesia, Malaysia, Philippines, Singapore, South Korea, and Taiwan). Participants received a survey hyperlink, an explanation of the study purpose, and access to the questionnaire after agreeing to participate. Each participant could respond only once and withdraw at any time by closing the browser. All responses were anonymous and confidential. The survey was open from 28 June to 30 November 2024.

The survey aimed to reach 1000 respiratory physicians, anticipating a 25% response rate to achieve a target of 250 responses. A sample size calculation using a 95% confidence level and a 5% margin of error indicated that 278 respondents were required. Considering the expected response rate, 1112 respiratory physicians were initially invited. Snowball sampling through questionnaire dissemination by recipients was permitted.

All questions were weighted equally to calculate the questionnaire completion rate. Responses with over 90% completion rate were considered valid. Statistical analyses were conducted using SPSS for Windows, Version 27.0 (IBM SPSS Inc, IL, USA). Descriptive statistics summarized demographic characteristics and responses. Likert scale responses were dichotomized (e.g. “most of the time” and “always” as “routine”, “never”, “sometimes”, and “half of the time” as “infrequent”) for analysis. Categorical variables were compared using chi-square or Fisher’s exact test. All clinically relevant variables examined in univariable analyses were entered into a global multivariable logistic regression model, followed by backward elimination (*p* < 0.05 removal) to derive a parsimonious final model. Collinearity between reported TB burden and TBP encounter frequency was assessed using Spearman correlation and linear regression diagnostics (variance inflation factor, VIF). Statistical significance was defined as *p* < 0.05. P-values were unadjusted due to the exploratory nature of the study, with significant findings considered hypothesis-generating. TB burden data for surveyed regions were retrieved from the WHO and the World Bank (Supplementary Table 1)^11,12^.

The primary objective was to determine the likelihood of considering TBP in patients presenting with unilateral pleural effusion. Secondary endpoints included factors contributing to this consideration, use of diagnostic tests to confirm TBP, and physicians’ perceptions of the diagnostic performance of various tests and biomarkers. Because diagnosing definite TBP can be challenging due to its heterogeneous presentation, the survey examined confidence in accepting a diagnosis of probable TBP, factors influencing the initiation of empirical anti-TB treatment, and barriers to TBP diagnosis using a clinical vignette.

Informed consent was obtained from all respondents. The study adhered to the Declaration of Helsinki. Ethical approval for questionnaire distribution in Hong Kong was obtained from the Chinese University of Hong Kong/New Territories East Cluster Clinical Research Ethics Committee (reference number 2023.666). The study was registered at ClinicalTrials.gov (NCT06390969) and supported by the Asian Pacific Society of Respirology Assembly Fund Project Grant.

## Results

A total of 418 responses were collected, with 414 (99.0%) valid responses analyzed.

### Characteristics of respondents

The characteristics and relevant clinical experience of respondents are detailed in Table 1. About half (49.0%) of the respondents had practised for over a decade. Respondents practised in high (25.1%) and intermediate (73.7%) TB burden regions, respectively. A high correlation was found between the perceived TB burden by respondents and the actual reported TB burden (Supplementary Table 2).

**Table 1:** Demographics and experience of the practice of respondents^1^.

Parameters	All respondents	Respondents from high TB burden regions	Respondents from low-to-intermediate TB burden regions	*p*-value^2^
Gender, n (%)				
Male Female Prefer not to disclose	234 (56.5) 172 (41.5) 8 (1.9)	71 (68.3) 32 (30.8) 1 (1.0)	163 (52.6) 140 (45.2) 7 (2.3)	0.019
Age, years, n (%)				
≤ 29 30–39 40–49 50–59 60–69 ≥ 70 Prefer not to disclose	35 (8.5) 178 (43.0) 129 (31.2) 65 (15.7) 3 (0.7) 2 (0.5) 2 (0.5)	4 (3.8) 55 (52.9) 32 (30.8) 12 (11.5) 0 (0.0) 1 (1.0) 0 (0.0)	31 (10.0) 123 (39.7) 97 (31.3) 53 (17.1) 3 (1.0) 1 (0.3) 2 (0.6)	0.104
Region of practice, n (%)				
Mainland China Hong Kong Taiwan Thailand India Philippines Malaysia South Korea Indonesia Singapore Australia Macau Others^3^	166 (40.1) 43 (10.4) 33 (8.0) 33 (8.0) 24 (5.8) 24 (5.8) 22 (5.3) 21 (5.1) 21 (5.1) 15 (3.6) 5 (1.2) 4 (1.0) 3 (0.7)	0 (0.0) 0 (0.0) 0 (0.0) 33 (31.7) 24 (23.1) 24 (23.1) 0 (0.0) 0 (0.0) 21 (20.2) 0 (0.0) 0 (0.0) 0 (0.0) 2 (0.7)	166 (53.5) 43 (13.9) 33 (10.6) 0 (0.0) 0 (0.0) 0 (0.0) 22 (7.1) 21 (6.8) 0 (0.0) 15 (4.8) 5 (1.6) 4 (1.3) 1 (0.8)	< 0.001
Experience of practice in respiratory medicine, n (%)^4^				
≤ 5 years 6–10 years 11–15 years 16–19 years ≥ 20 years	110 (26.6) 101 (24.4) 87 (21.0) 43 (10.4) 73 (17.6)	40 (38.5) 25 (24.0) 24 (23.1) 6 (5.8) 9 (8.7)	70 (22.6) 76 (24.5) 63 (20.3) 37 (11.9) 64 (20.6)	0.002
Major site of practice, n (%)				
Private institute (non-teaching hospital) Public institute (non-teaching hospital) University-affiliated or teaching hospital Others^5^	40 (9.7) 133 (32.1) 238 (57.5) 3 (0.7)	21 (20.2) 16 (15.4) 65 (62.5) 2 (1.9)	19 (6.1) 117 (37.7) 173 (55.8) 1 (0.3)	< 0.001
Frequency of encountering patients with new-onset unilateral pleural effusion, n (%)				
Never < 5 / year 5–10 / year 1–4 / month 5–10 / month > 10 / month	3 (0.7) 25 (6.0) 65 (15.7) 146 (35.3) 90 (21.7) 85 (20.5)	0 (0.0) 0 (0.0) 5 (4.8) 31 (29.8) 34 (32.7) 34 (32.7)	3 (1.0) 25 (8.1) 60 (19.4) 115 (37.1) 56 (18.1) 51 (16.5)	< 0.001
Proportion of TBP among patients with new-onset unilateral pleural effusion, n (%)				
< 5% 5–10% 11–25% 26–50% > 50%	75 (18.1) 89 (21.5) 94 (22.7) 112 (27.1) 44 (10.6)	6 (5.8) 15 (14.4) 19 (18.3) 39 (37.5) 25 (24.0)	69 (22.3) 74 (23.9) 75 (24.2) 73 (23.5) 19 (6.1)	< 0.001

^1^Row and column percentages may not sum to exactly 100% due to rounding.

^2^Comparison between respondents from high TB burden regions and those from low-to-intermediate TB burden regions.

^3^Other countries include Myanmar (1), Vietnam (1) and Türkiye (1).

^4^Includes the training period.

^5^Other sites of practice include all of the above (1), private and teaching hospitals (1) and public and teaching hospitals (1).

### Investigating new-onset unilateral pleural effusion

In the evaluation of new-onset unilateral pleural effusion, 80.4% of respondents considered TBP as a differential diagnosis. This was associated with frequent encounters of new-onset unilateral pleural effusion and TBP, and intermediate regional TB burden, as shown by multivariable regression analysis (Table 2 and Supplementary Table 3). Reported TB burden and frequency of encountering TBP showed moderate correlation in this regression model (ρ = 0.333, *p* < 0.001). Clinical exposure variables showed minimal collinearity (VIF for TB burden 1.09–1.22; maximal VIF for TBP frequency 1.57), supporting model stability.

**Table 2:** Multivariable analysis of factors that correlate with the consideration of TBP as a differential diagnosis.

Variables	Odds ratio (95% CI)
Reported intermediate TB burden	2.26 (1.25–4.07)
Encounter new-onset unilateral pleural effusion 5–10 times / year	3.18 (1.25–8.10)
Encounter new-onset unilateral pleural effusion 1–4 times / month	1.66 (0.95–2.89)
11–25% of new-onset unilateral pleural effusion being TBP	2.74 (1.39–5.42)
26–50% of new-onset unilateral pleural effusion being TBP	2.95 (1.53–5.71)
> 50% of new-onset unilateral pleural effusion being TBP	16.01 (3.52–72.89)

All clinically relevant variables from univariable analysis were entered into a global multivariable logistic regression model, followed by backward elimination (*p* < 0.05 removal) to derive a parsimonious final model. The final model demonstrated good classification accuracy (80.4% correctly classified) with good fit (Hosmer and Lemeshow Test of χ²=2.8, df = 8, *p* = 0.948).

CI: confidence interval; TB: tuberculosis; TBP: tuberculous pleuritic.

Practice variation was observed in the investigative approach (Tables 3 and 4, Supplementary Table 4). Several TBP-related tests were reported as unavailable in respondents’ institutions (Supplementary Table 5). Pleural fluid was routinely sent for MTB culture by 57.7% of respondents, for AFB smear by 73.9%, for ADA by 81.6%, concurrent parietal pleural biopsy for histology by 26.6%, and pleural tissue for MTB culture by 38.2%. Only 16.4% of respondents sampled pleural tissue for histology and MTB culture simultaneously. Respondents from low-to-intermediate TB burden regions were more likely to request pleural fluid ADA, AFB smear, MTB culture, and sputum for AFB smear and/or MTB culture. Respondents who commonly considered TBP were more likely to request pleural fluid ADA, MTB PCR, and pleural tissue for histology.

**Table 3:** Pleural investigations that are commonly ordered during the first diagnostic thoracentesis for new-onset unilateral pleural effusion, with comparison made between reported high versus low or intermediate TB burdens^1,2^.

Pleural investigations	Respondents answering “most of the time” or “always” (*n* = 414)	Respondents answering “not available” (*n* = 414)	Reported high TB burden^3^	Reported low / intermediate TB burden^3^	*p*-value^4^
White blood / nucleated cell differential count, n (%)	363 (87.7)	17 (4.1)	96 / 104 (92.3)	267 / 293 (91.1)	0.711
Light’s criteria (total protein and LDH), n (%)	393 (94.9)	4 (1.0)	97 / 103 (94.2)	296 / 307 (96.4)	0.323
ADA, n (%)	338 (81.6)	17 (4.1)	62 / 95 (65.3)	276 / 302 (91.4)	< 0.001
AFB smear, n (%)	306 (73.9)	5 (1.2)	53 / 104 (51.0)	253 / 305 (83.0)	< 0.001
MTB culture, n (%)	239 (57.7)	33 (8.0)	44 / 99 (44.4)	195 / 282 (69.1)	< 0.001
MTB PCR, including GeneXpert or Xpert Ultra, n (%)	186 (44.9)	22 (5.3)	57 / 103 (55.3)	129 / 289 (44.6)	0.062
Pleural biopsy for histology, n (%)	110 (26.6)	10 (2.4)	20 / 100 (20.0)	90 / 304 (29.6)	0.061
Pleural biopsy for MTB culture, n (%)	158 (38.2)	26 (6.3)	41 / 100 (41.0)	117 / 288 (40.6)	0.948
Sputum for AFB smear and/or MTB culture, n (%)	315 (76.1)	9 (2.2)	70 / 104 (67.3)	245 / 301 (81.4)	0.003

^1^The full table is presented in the Supplementary Materials (Supplementary Table 4).

^2^Row and column percentages may not sum to exactly 100% due to rounding.

^3^Percentages are calculated with respondents reporting that corresponding tests are available as the denominator.

^4^Comparison between reported high and low / intermediate TB burden for respondents’ regions of practice.

ADA: adenosine deaminase; AFB: acid-fast bacilli; LDH: lactate dehydrogenase; MTB: Mycobacterium tuberculosis; PCR: polymerase chain reaction.

**Table 4:** Pleural investigations that are commonly ordered during the first diagnostic thoracentesis for new-onset unilateral pleural effusion, with comparison made between respondents based on their likelihood of considering TBP as a differential diagnosis^1^.

Pleural investigations	Respondents “most of the time” or “always” consider TBP as a differential diagnosis^2,3^	Respondents “never”, “sometimes” or “about half of the time” consider TBP as a differential diagnosis^2,3^	*p*-value
White blood / nucleated cell differential count, n (%)	299 / 320 (93.4)	64 / 77 (83.1)	0.004
Light’s criteria (total protein and LDH), n (%)	322 / 330 (97.6)	71 / 80 (88.8)	< 0.001
ADA, n (%)	287 / 318 (90.3)	51 / 79 (64.6)	< 0.001
AFB smear, n (%)	249 / 328 (75.9)	57 / 81 (70.4)	0.303
MTB culture, n (%)	187 / 301 (62.1)	52 / 80 (65.0)	0.637
MTB PCR, including GeneXpert or Xpert Ultra, n (%)	163 / 313 (52.1)	23 / 79 (29.1)	< 0.001
Pleural biopsy for histology, n (%)	101 / 325 (31.1)	9 / 79 (11.4)	< 0.001
Pleural biopsy for MTB culture, n (%)	129 / 310 (41.6)	29 / 78 (37.2)	0.476
Sputum for AFB smear and/or MTB culture, n (%)	269 / 325 (82.8)	46 / 80 (57.5)	< 0.001

^1^The full table is presented in the Supplementary Materials (Supplementary Table 4).

^2^Row and column percentages may not sum to exactly 100% due to rounding.

^3^Percentages are calculated with respondents reporting that corresponding tests are available as the denominator.

ADA: adenosine deaminase; AFB: acid-fast bacilli; LDH: lactate dehydrogenase; MTB: Mycobacterium tuberculosis; PCR: polymerase chain reaction.

### Physician confidence in TBP diagnosis

Regarding confidence in diagnosing TBP and initiating empirical anti-TB therapy, 60.9% of respondents required microbiological or histological evidence, 37.4% would initiate treatment based on clinical presentation alone, and 1.7% would diagnose TBP clinically without pleural fluid analysis. Respondents preferring definitive TBP diagnosis were associated with low-to-intermediate reported TB burden in their region of practice (68.4% vs. 38.5%, *p* < 0.001), and preference for pleural biopsy for histology (Supplementary Table 6).

### Understanding of TBP-related diagnostic tests and the utility of pleural fluid biomarkers

**Fig. 1:**
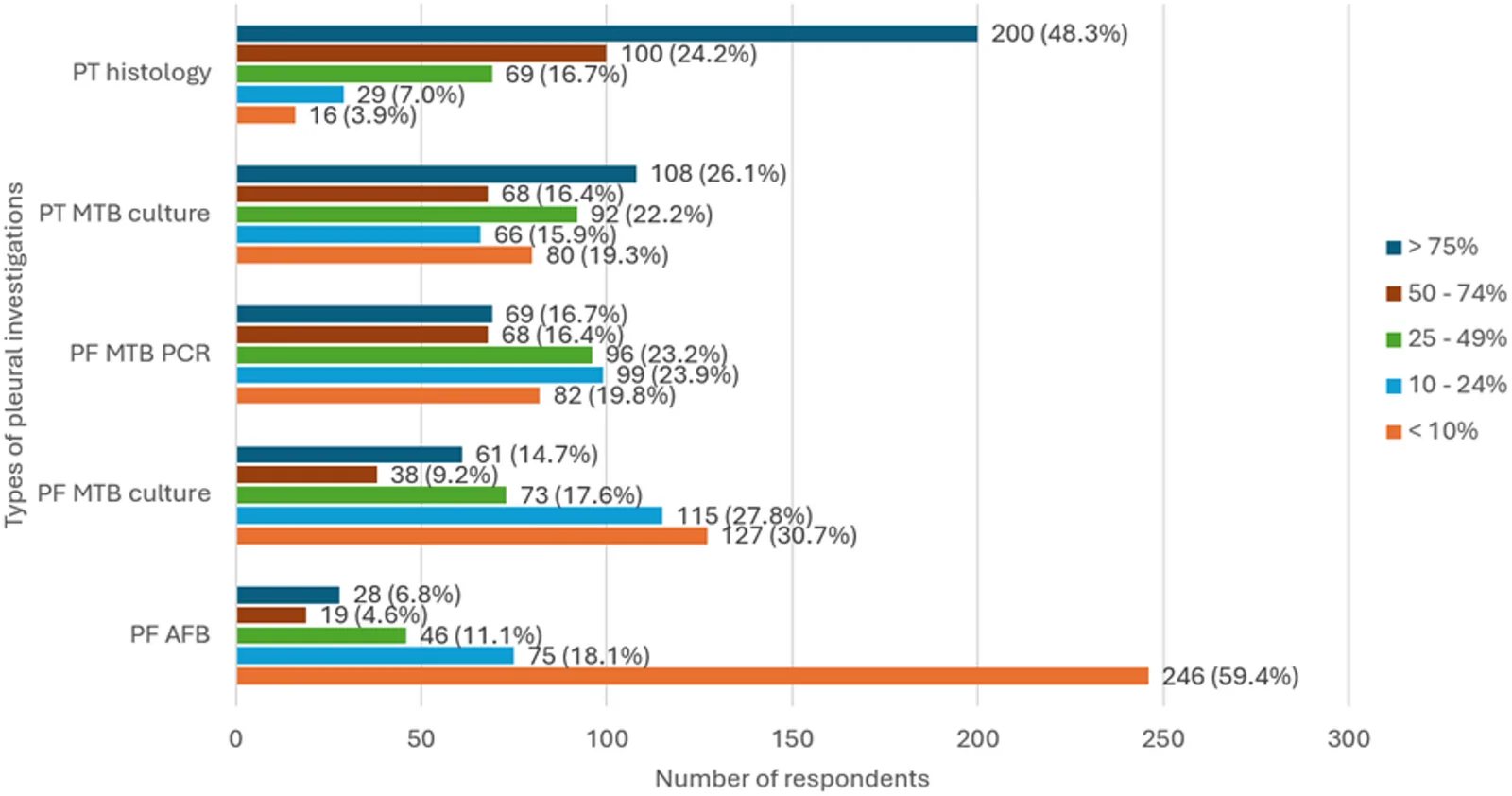
Perception of the diagnostic accuracy of different tests for diagnosing TBP. This figure shows the distribution of perceived diagnostic accuracy (< 10%, 10–24%, 25–49%, 50–74%, and > 75%) for various TBP-related investigations among respondents. Nearly half (200, 48.3%) of respondents perceived that pleural tissue histology has the highest diagnostic accuracy (> 75%) for TBP. 168 (40.6%) and 176 (42.5%) of respondents perceived that pleural fluid AFB stain and MTB culture have a diagnostic accuracy of ≥ 10% and ≥ 50%, respectively. Percentages may not sum to exactly 100% due to rounding. AFB: acid-fast bacilli; MTB: Mycobacterium tuberculosis; PCR: polymerase chain reaction; PF: pleural fluid; PT: pleural tissue.

More than half of the respondents (59.4%) perceived pleural fluid AFB sensitivity as < 10%, whereas 23.9%, 33.1%, 42.5%, and 72.5% perceived sensitivity ≥ 50% for pleural fluid MTB culture, MTB PCR, pleural tissue MTB culture, and histology showing caseating granulomas, respectively. These perceptions were independent of regional TB burden (Fig. 1 and Supplementary Table 7). Nearly all respondents (97.8%) recognized elevated pleural fluid ADA levels as associated with TBP, with a lower diagnostic cutoff of 38.2 ± 14.8 U/L. Among pleural fluid biomarkers (e.g. interferon-gamma, interleukin-2 or interleukin-17), respondents showed greatest confidence in ADA for TBP diagnosis (Supplementary Table 8).

### Management of TBP

**Fig. 2:**
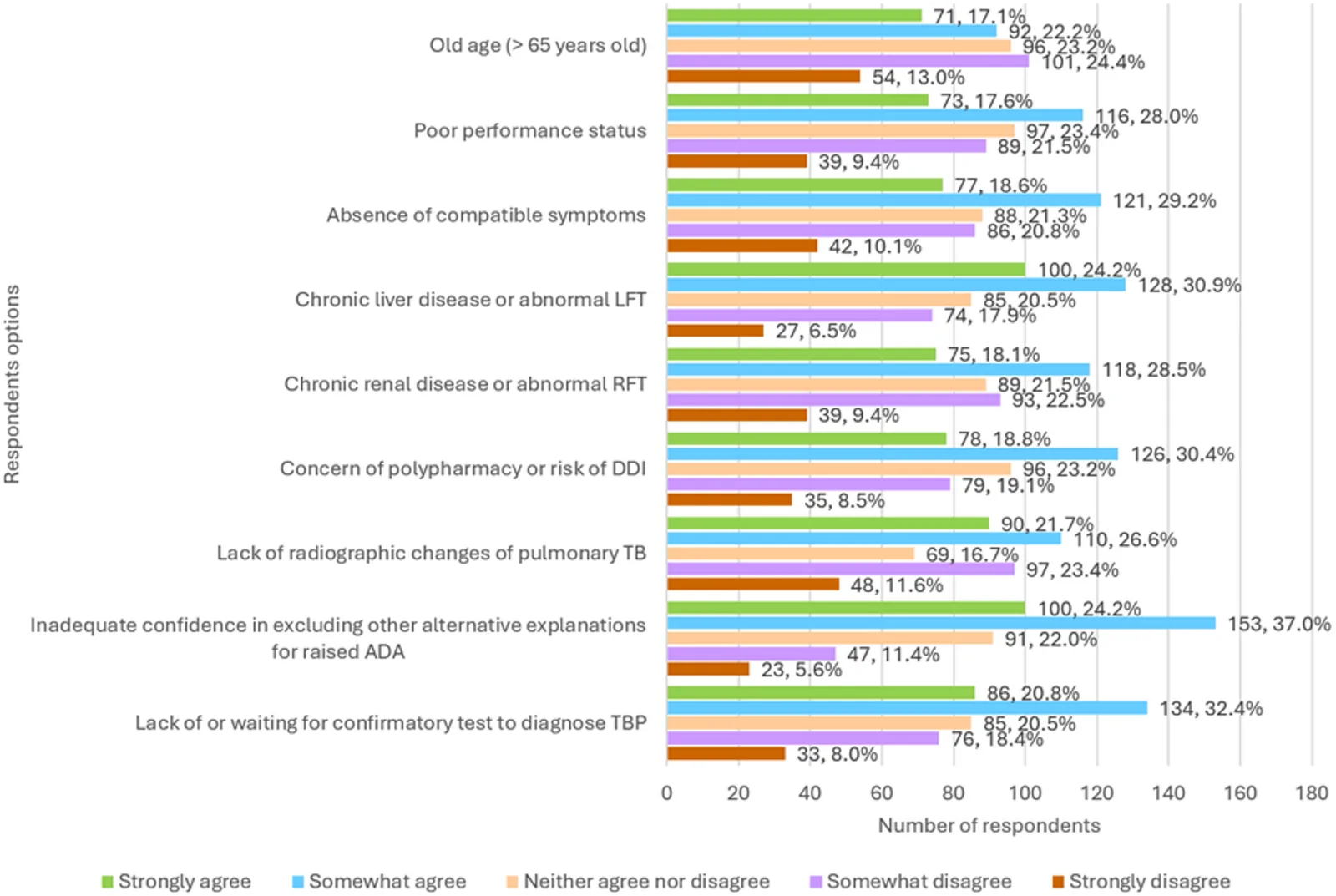
Factors that may affect the decision of empirical anti-TB treatment in patients with probable TBP. This figure shows the distribution of respondents who considered clinical factors when deciding on empirical anti-TB treatment for patients with probable TBP, as described in the clinical vignette. Age > 65 years, lack of radiographic pulmonary abnormalities suggestive of pulmonary TB and concomitant renal disease or abnormal renal function were the three most common reasons. Percentages may not sum to exactly 100% due to rounding. ADA: adenosine deaminase; DDI: drug-drug interaction; LFT: liver function test; RFT: renal function test; TB: tuberculosis; TBP: tuberculous pleuritic.

Factors that may delay the initiation of empirical anti-TB treatment for probable TBP, based on clinical vignette responses, include age > 65 years (37.4%), lack of radiographic pulmonary abnormalities suggestive of pulmonary TB (35.0%), and concomitant renal disease or abnormal renal function (31.9%) (Fig. 2). Respondents from high TB burden regions were less likely to delay treatment due to lack of compatible symptoms but more likely to delay treatment if lack or waiting for confirmatory tests (e.g. positive MTB culture or PCR result) (Supplementary Table 9).

Pleuroscopy was preferred by 49.5% where pleural biopsy was needed. Based on clinical experience, 77.8% of respondents reported revising probable TBP diagnosis after empirical anti-TB treatment failure, with higher rates among those from high TB burden regions (80.8% vs. 76.8%, *p* = 0.021). Practice regarding systemic corticosteroids and therapeutic pleural drainage for confirmed TBP also varied (Supplementary Table 10).

### Barriers to TBP diagnosis

**Fig. 3:**
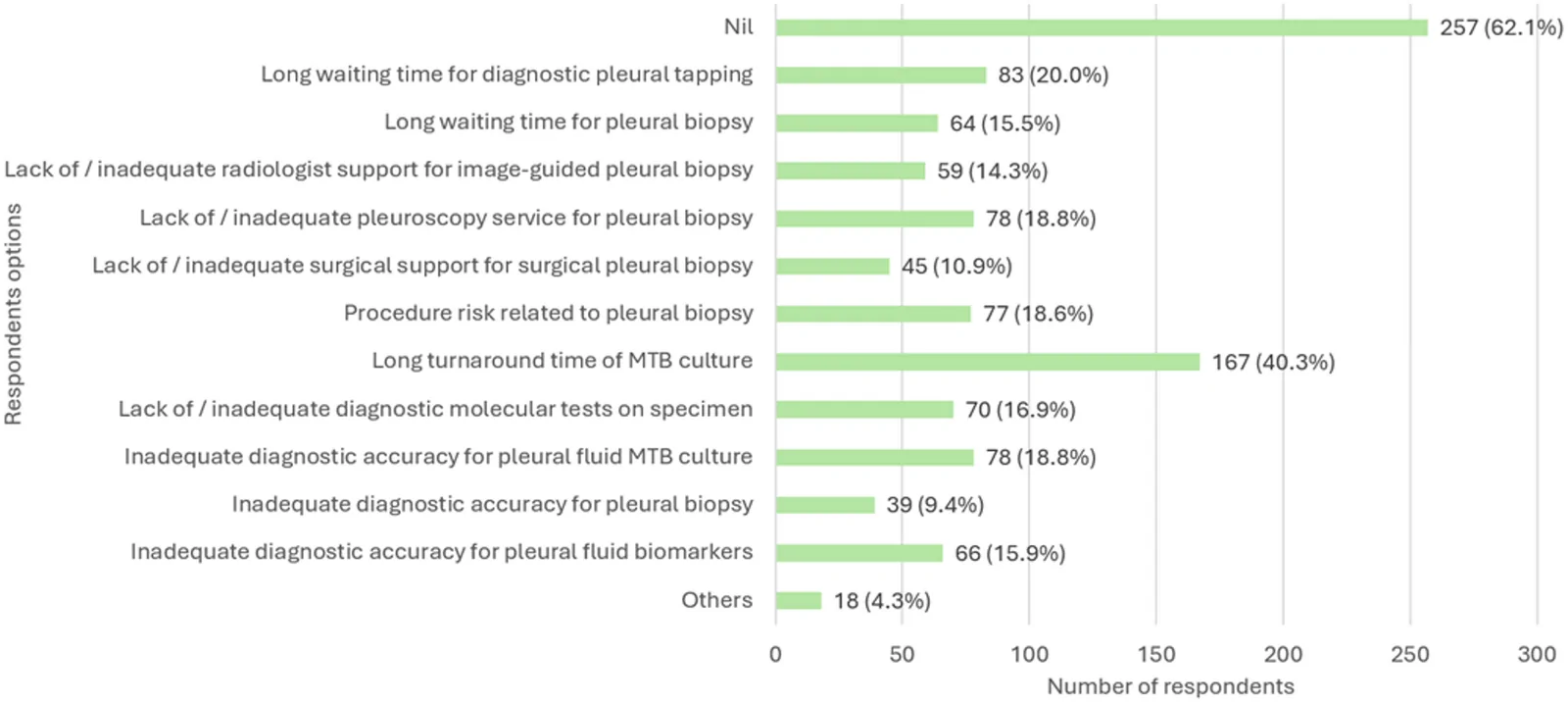
Perceived difficulties and barriers in diagnosing TBP among respondents. This figure shows the perceived difficulties and barriers in diagnostic TBP among respondents. Barriers were reported by 37.9% respondents. The limitation of pleural biopsy and the suboptimal diagnostic accuracy of various TBP-related investigations were considered important barriers for achieving timely and accurate diagnosis of TBP. Others include patient not consenting to pleural biopsy (5), inadequate accuracy or delayed in reporting of pleural fluid MTB PCR (3), lack or high cost of pleural fluid ADA (3), delay in referral/lack of suspicion (2), limitation due to healthcare policy (2), uncertain diagnosis due to negative initial pleural fluid workup (2), lack of pleural biopsy service (1). Percentages may not sum to exactly 100% due to rounding. ADA: adenosine deaminase; CT: computed tomography; MTB: Mycobacterium tuberculosis; PCR: polymerase chain reaction; TBP: tuberculous pleuritic.

Barriers were reported by 37.9% respondents, including long turnaround time for MTB culture (40.3%), long waiting time for diagnostic thoracentesis (20.0%), lack of pleuroscopy expertise (18.8%), and high reported complications of pleural biopsy (18.6%) (Fig. 3). Pleural fluid ADA and tissue histology results were available within five working days for 78.3% and 53.4% of respondents, respectively. Among pleural biopsy services, pleuroscopy (69.3%) was most available, while surgical biopsy had the longest waiting time (median 5 days). No pleural biopsy services were available for 6.8% of respondents (Supplementary Table 11).

## Discussion

This survey documents self-reported practice patterns and perceptions of test performance among 414 respiratory physicians. Most respondents (80.4%) practising frequently considered TBP when evaluating patients with unilateral pleural effusion. This reflects TBP as a leading cause of non-malignant exudative pleural effusion^13–19^. Clinical judgement was associated with regional TB burden and frequency of encountering TBP or pleural effusion, resulting in diverse practice patterns that reflect local epidemiology and available resources. Variability was also observed in the interpretation of diagnostic tests and confidence in initiating empirical anti-TB treatment.

Heterogeneity exists in the diagnostic approach for new-onset pleural effusion. Pleural fluid ADA, AFB smear and MTB culture are not routinely ordered, and closed pleural biopsy during the initial thoracentesis was performed by only one-quarter of respondents. This practice contrasts with the British Thoracic Society (BTS) guideline, which recommends pleural fluid ADA testing and pleural tissue sampling for MTB culture in suspected TBP cases^20^. Since the sensitivity of clinical prediction rules suspecting TBP varies geographically and equals pleural fluid ADA at best^21^, a standardized panel of pleural tests, including microbiological, histological tests and molecular analyses, should be used whenever available to diagnose or exclude TBP.

Heterogeneity also extended to diagnostic confidence in accepting definite versus probable TBP diagnoses to initiate anti-TB treatment. Practical considerations, such as prolonged MTB culture turnaround times and delays in accessing biopsy services, likely compelled physicians to accept probable diagnoses rather than pursue further invasive investigations. The survey also revealed marked discrepancies in perceived sensitivity of TBP tests, with many respondents overestimating pleural fluid AFB and MTB culture sensitivity^9^. Given the paucibacillary nature and diverse presenting features of TBP, frequent false-negative results may mislead clinicians and limit further diagnostic evaluation, potentially missing TBP diagnoses and increasing recurrence risk^22–24^.

Regional differences in TB burden contributed to this heterogeneity. More frequent TBP encounters among respondents from TB-endemic regions likely increased vigilance. Respondents from high TB burden regions showed greater confidence in accepting probable TBP and initiating empirical treatment. However, this approach risks incorrect diagnoses, requiring revisions of the diagnosis after failure of empirical anti-TB treatment. High prevalence of drug-resistant TB and inadequate access to timely pleural services in TB-endemic regions may contribute. This finding aligns with an Indian cohort where 52.6% of patients with unexplained pleural effusion received initial anti-TB treatment, but only 11.8% had confirmed TBP^15^.

Several areas warrant further study. Targeted recommendations accounting for regional endemicity and resource constraints should guide clinical decisions in TB-endemic regions. Although the BTS guideline mentions biomarkers (ADA and/or IFN-gamma) and pleural tissue sampling for MTB culture, it provides no guidance on interpreting unexpected results when TBP is strongly suspected in TB-prevalent regions^20^. TBP is also absent from the latest European Respiratory Society statement on benign pleural effusions in adults^25^. Variable pleural fluid ADA cutoffs (36–65 U/L) have been reported^26^, consistent with our findings. The survey assessed physicians’ confidence in ADA (their preferred biomarker) and the factors influencing treatment decisions according to the ADA levels. MTB PCR (Xpert MTB/RIF Ultra), recommended by the WHO^27^, shows 68% pooled sensitivity but reduced performance in culture-negative TBP cases, necessitating careful interpretation of negative results^10^. Variable use of systemic corticosteroids and therapeutic thoracentesis has been reported previously^28–33^. These inadequacies in guidelines and the diversity of practice underscore the need for prospective studies and diagnostic algorithms tailored to TB-prevalent regions.

Several limitations should be acknowledged. Convenience sampling introduced bias due to underrepresentation of low TB burden regions (e.g. Australia) and over-representation of mainland China and Hong Kong (50.5% combined), inadequate coverage of all respiratory physicians within regions, and uneven practice experience distribution. The study findings reflect patterns within this respondent cohort rather than regional norms. Self-reports may differ from actual behaviour due to recall bias or idealized reporting. Misdiagnosis rates and test accuracy estimates reflect subjective experience, not objective validation, and require confirmation by prospective audit or registry data. Dichotomization of Likert scale responses prioritized clinical relevance but lost ordinal granularity. The multivariable model included moderately collinear predictors (TB burden and TBP encounter frequency, ρ = 0.333, VIF ≤ 1.57), which may inflate standard errors. The study did not fully evaluate sputum workup (AFB smear, MTB culture and MTB PCR) or computed tomography, which help differentiate TBP from malignancy. New-onset pleural effusion with a possible malignant nature may prompt more extensive investigations, which was not explored. Finally, a single non-interactive clinical vignette may not capture the heterogeneous presentation of TBP, though it facilitated pragmatic decision-making. These factors limit generalizability beyond the vignette and surveyed cohort.

## Conclusion

This survey of 414 respiratory physicians reveals marked variability in diagnostic test selection for new-onset unilateral pleural effusion, confidence thresholds for empirical treatment for TBP, and procedural availability across different institutions of the respondents. These findings underscore the need for standardized practices to improve TBP diagnosis, particularly where TB burden is high. Aligning diagnostic and management strategies with local resource constraints and TB epidemiology emphasizes the importance of TBP awareness and appropriate pleural investigations. Addressing these disparities remains crucial for better patient outcomes.

## Supplementary Information

Below is the link to the electronic supplementary material.

Supplementary Material 1

Supplementary Material 2

## Data Availability

The data that support the findings of this study are available from the corresponding author, K. K. P. CHAN, upon reasonable request.
