# Supplementary material for: A survey of respiratory physicians on investigating and managing tuberculous pleuritis
Source: Sci Rep. 2026 Jun 10;16:26533. doi: 10.1038/s41598-026-57407-3 (PMC13503735; doi:10.1038/s41598-026-57407-3)
Supplement: Supplementary file 1 [file 41598_2026_57407_MOESM2_ESM.pdf]

A survey on the practice and interpretation of various diagnostic tests for tuberculous pleuritis among respiratory physicians

Introduction:
We are a group of respiratory physicians from the Asia-Pacific region.

You are invited to participate in a research study on '**A survey on the application and interpretation of diagnostic tests for tuberculous pleuritis among respiratory physicians'**. You will be asked to complete an online survey to evaluate your perception and acceptance of diagnostic tests for tuberculous pleuritis. It takes about 10 minutes to complete the questionnaire. Please select the answers that best fit your understanding and current clinical practice during the process.

Your participation in this study is completely voluntary. The study is anonymous, and each individual can participate only once. The information you provide will be kept confidential and used for research purposes only. Possible dissemination outlets include conferences, presentations and/or publications in scientific journals. No identifiable personal information will appear in any future presentations or publications. During the survey, you may withdraw anytime by simply closing the browser, and in such case, your response will not be recorded.

There are no clinical risks to individuals participating in this study. Although the study may not benefit you directly, your response could help us understand the practices among respiratory physicians for ordering and interpreting pleural fluid analysis. The information gathered will also guide subsequent research in this field.

You are assured that refusal or withdrawal from the study will not have any consequence. For questions about the present study, you may contact our principal investigator, Dr Ken KP Chan (email: chankapang@cuhk.edu.hk or by phone: +852 3505 3532). You can also call The Joint CUHK-NTEC Clinical Research Ethics Committee at +852 3505 3935 for study subjects' rights-related questions. We thank you for your support and participation in this study.

Response:
I have read and understood the above consent form. I certify that I am 18 years old or older, and by clicking the "**I am willing to participate in this study** " button to enter the survey, I indicate my willingness to participate voluntarily.

- I am willing to participate in this study, route to the survey page (English).
- I do not wish to participate in this study, route to the end page.

(the above will be displayed on the first page of the online questionnaire)

(English version)

Part 1. Background information

1. What is your **gender**?
   A. Male
   B. Female
   C. Prefer not to disclose
2. What is your **age group**?
   A. ≤ 29
   B. 30 – 39
   C. 40 – 49
   D. 50 – 59
   E. 60 – 69
   F. ≥ 70
   G. Prefer not to disclose
3. Where is your **region of practice**?
   A. Australia
   B. Hong Kong SAR
   C. India
   D. Japan
   E. Mainland China
   F. Macau SAR
   G. Malaysia
   H. Myanmar
   I. Philippines
   J. Singapore
   K. Taiwan
   L. Thailand
   M. South Korea
   N. Vietnam
   O. Other, please specify
4. How long have you been practising in **respiratory medicine**, including the training period?
   A. ≤ 5 years
   B. 6 – 10 years
   C. 11 – 15 years
   D. 16 – 19 years
   E. ≥ 20 years
5. Which of the following areas best describes the majority of your practice?

A. Private hospital (non-teaching hospital)
B. Public hospital (non-teaching hospital)
C. University affiliated or teaching hospital
D. Others, please specify

Part 2. Usual practice of investigating new-onset unilateral pleural effusion

6. How frequently do you encounter patients with **new-onset unilateral pleural effusion**?
   A. Never
   B. Less than 5 patients **per year**
   C. 5 to 10 patients **per year**
   D. 1 to 4 patients **per month**
   E. 5 to 10 patients **per month**
   F. More than 10 patients **per month**
7. Among patients with new-onset unilateral pleural effusion, what proportion of them have **tuberculous pleuritis**?
   A. < 5%
   B. 5 – 10%
   C. 11 – 25%
   D. 26 – 50%
   E. > 50%
8. What is your **perceived tuberculosis burden** in your region of practice?
   A. Low
   B. Intermediate
   C. High

Questions 9 to 22. For patients with new onset unilateral pleural effusion, which of the following pleural fluid tests would you commonly order at the first diagnostic thoracentesis (multiple options may be chosen)?

	Never	Sometimes	About half the time	Most of the time	Always	Test not available in my institute
	A	B	C	D	E	F
9. Red cell count						
10. White blood / nucleated cell total						

cell count						
11. White blood / nucleated cell differential count						
12. Light's criteria (total protein, lactate dehydrogenase [LDH])						
13. Albumin						
14. Adenosine deaminase (ADA)						
15. pH						
16. Glucose						
17. Bacterial culture (in plain bottle)						
18. Bacterial culture (in culture bottle)						
19. Acid-fast bacilli (AFB) smear						
20. *Mycobacterium tuberculosis* (MTB) culture						
21. MTB polymerase chain reaction (PCR), including GeneXpert or Xpert Ultra						
22. Cytology, paraffin embedded pathological analysis						

23. Others, please specify

24. How often do you consider TB pleuritis as one of the differential diagnoses?
    A. Never
    B. Sometimes
    C. About half of the time
    D. Most of the time
    E. Always

Questions 25 to 28. For patients with new-onset unilateral pleural effusion…

	Never	Sometimes	About half of the time	Most of the time	Always	Not available
	A	B	C	D	E	F
25. How often do you order pleural fluid **ADA** levels?						
26. How often do you send pleural tissue (e.g. bedside blinded pleural biopsy or image-guided pleural biopsy) for **histology** during the initial diagnostic pleural tapping?						
27. How often do you send pleural tissue (e.g. bedside blinded pleural biopsy or image-guided pleural biopsy) for **MTB culture** during the initial pleural biopsy?						
28. How often do you send **sputum** for acid-fast bacilli smear and/or MTB						

culture?						

29. What is the average turnaround time (**in working days**) for the **pleural fluid ADA** test in your institute?
    A. Within 2 working days
    B. 3 to 5 working days
    C. 6 to 10 working days
    D. More than 10 working days
    E. Test not available in my institute
30. What is the average turnaround time (**in working days**) for the **pleural tissue** histology report in your institute?
    A. Within 2 working days
    B. 3 to 5 working days
    C. 6 to 10 working days
    D. More than 10 working days
    E. Test not available in my institute
31. Does your institute have the following services (multiple options may be chosen)? What is their **average waiting time**?
    A. **Non-ultrasound guided pleural biopsy** by <u>respiratory physicians</u>, waiting time (in working days): ___
    B. **Real-time ultrasound-guided pleural biopsy** by <u>respiratory physicians</u>, waiting time (in working days): ___
    C. **Real-time image-guided** (CT or ultrasound) **pleural biopsy** by <u>radiologists</u>, waiting time (in working days): ___
    D. **Pleuroscopy** (medical thoracoscopy) **guided pleural biopsy** by <u>respiratory physicians</u>, waiting time (in working days): ___
    E. **Surgical pleural biopsy** by <u>thoracic surgeon</u>, waiting time (in working days): ___
    F. None of the above

Part 3. Perception of the diagnostic accuracy of different tests for diagnosing tuberculous pleuritis (TBP) by physicians

Questions 32 – 36. What is the sensitivity of the following tests in diagnosing TBP according to your understanding?

A. < 10%
B. 10 - 24%
C. 25 - 49%
D. 50 - 74%
E. ≥ 75%

	A	B	C	D	E
32. Pleural fluid AFB smear					
33. Pleural fluid MTB culture					
34. Pleural fluid MTB PCR					
35. Pleural tissue MTB culture					
36. Pleural tissue histology					

37. Which conditions can **potentially elevate** the pleural fluid **ADA levels**? Please choose all that apply.
    - A. Tuberculous pleuritis
    - B. Pleural infection (complicated parapneumonic effusion, empyema)
    - C. Malignant pleural effusion due to solid cancers
    - D. Heart failure
    - E. Lymphoma
    - F. None of the above
38. At what **cut-off level of pleural fluid ADA** would you consider empirical TB treatment in the absence of microbiological or histological evidence, assuming a compatible clinical history and radiological features?
    - A. Upper limit: 0 – 100 (in scale points), or not applicable
    - B. Lower limit: 0 – 100 (in scale points), or not applicable
39. Other than ADA, what pleural fluid biomarkers do you use to diagnose TBP in your institute (more than one option may be chosen)?
    - A. Nil others (to question 41)
    - B. **Pleural fluid** interferon-gamma (**IFN-gamma**), please specify the cutoff if any
    - C. **Pleural fluid** tumour necrosis factor-alpha (**TNF-alpha**), please specify the cutoff if any
    - D. **Pleural fluid** interferon-gamma release assay (**IGRA**), please specify the cutoff if any
    - E. **Pleural fluid interleukins** (e.g. IL-2, IL-27), please specify the cutoff if any
    - F. **Pleural fluid LDH / ADA ratio**, please specify the cutoff if any
    - G. Others, please specify
40. If you could select amongst a variety of pleural fluid biomarkers for the diagnosis of TBP at your institute, which one would you have the **most confidence** in, assuming compatible clinical findings?
    - A. ADA
    - B. Interferon-gamma (IFN-gamma)

C. Tumour necrosis factor-alpha (TNF-alpha)
D. Interferon-gamma release assay (IGRA)
E. Interleukins (e.g. IL-2, IL-27), please specify the type
F. LDH / ADA ratio
G. Others, please specify

41. Which of the following **best describes the most common way** of diagnosing TBP in your practice?
    A. By compatible clinical features only (new onset unilateral pleural effusion, features of pulmonary tuberculosis or successful trial of anti-tuberculous treatment), **without performing pleural fluid analysis**
    B. By compatible clinical features (as above) and suggestive pleural fluid results, **without the need to wait for** positive pleural fluid / tissue microbiological or histological findings
    C. By positive pleural fluid / tissue microbiological tests (AFB stain, MTB culture, or MTB PCR), or pleural tissue histological findings (e.g. necrotizing granulomatous inflammation), **regardless** of other clinical features or pleural fluid analysis results

Part 4. The decision to start empirical anti-tuberculosis treatment

Case example:

A patient presents with new onset unilateral pleural effusion, with pleural fluid analysis showing a lymphocytic nature (lymphocyte > 80%), exudative according to the Light's criteria, negative AFB stain and nil malignant cells found on cytological examination. **The patient has a pleural fluid ADA level that slightly above the cut-off range you chose in Question 38. You are suspecting the patient may have tuberculous pleuritis.**

Questions 42 – 50. In the presence of the following clinical conditions, you would be **inclined NOT TO start empirical anti-TB treatment**:

A. Strongly agree
B. Somewhat agree
C. Neither agree nor disagree
D. Somewhat disagree
E. Strongly disagree

	A	B	C	D	E
42. Old age (> 65 years old)					

43. Poor performance status					
44. Absence of compatible symptoms, e.g. fever, pleurisy					
45. Presence of chronic liver disease or abnormal liver function test					
46. Presence of chronic renal disease or abnormal renal function test					
47. Concern of polypharmacy and risk of drug-drug interaction					
48. Lack of radiographic changes suggestive of pulmonary tuberculosis (e.g. consolidation, fibronodular infiltrates or cavitary lesions) in the lungs by chest images					
49. Inadequate confidence in excluding other alternative explanations for raised ADA					
50. Lack of or waiting for confirmatory test to diagnose TBP (e.g. pleural fluid or pleural tissue MTB culture / MTB PCR, or compatible histological findings)					

51. If there are concerns other than those stated above (questions 42 to 50) that affect your decision to start anti-tuberculosis treatment, please specify.
52. If a pleural biopsy is needed in this patient, what is your **usual practice**?
    A. **Non-ultrasound guided** pleural biopsy by respiratory physicians
    B. **Real-time ultrasound-guided** pleural biopsy by respiratory physicians
    C. **Real-time image-guided (CT or ultrasound)** pleural biopsy by radiologists
    D. **Pleuroscopy (medical thoracoscopy)** guided pleural biopsy by respiratory physicians
    E. **Surgical** pleural biopsy by thoracic surgeon
    F. Pleural biopsy is **not available** in my institute
53. From your experience, how many of your patients required a **revision of diagnosis for pleural effusion** due to response failure to empirical anti-TB treatment?
    A. None. I never start empirical anti-TB treatment without a definite diagnosis based on pleural fluid / tissue microbiological or histological findings
    B. 1 to 9%
    C. 10 to 24%
    D. 25 to 49%
    E. 50 to 74%

F. > 75%

54. In addition to anti-TB treatments, do you prescribe **corticosteroids for confirmed TBP** in your practice?
    A. Yes
    B. No
55. If there is residual pleural effusion after diagnosing TBP, would you consider drainage of the effusion in addition to anti-TB treatment?
    A. **No**. To continue with the anti-TB treatment
    B. Yes, I would drain the effusion even if it **occupies < 25%** of the hemithorax
    C. Yes, I would drain the effusion if it **occupies > 25%** of the hemithorax
    D. Yes, I would drain the effusion if it **occupies > 50%** of the hemithorax
    E. Yes, I would drain the effusion **according to patient's symptom only** (e.g. shortness of breath, fever), regardless of effusion size

Part 5. Other information

56. What are the perceived difficulties and barriers in diagnosing TBP in your practice (multiple options may be chosen)?
    A. Nil
    B. Long waiting time for diagnostic pleural tapping
    C. Long waiting time for pleural biopsy
    D. Lack of / inadequate radiologist support for image-guided (CT or ultrasound) pleural biopsy service
    E. Lack of / inadequate pleuroscopy service for pleural biopsy
    F. Lack of / inadequate surgical support for surgical pleural biopsy
    G. Procedure risk related to pleural biopsy
    H. Long turnaround time of MTB culture
    I. Lack of / inadequate diagnostic molecular tests (e.g. MTB PCR) on specimen
    J. Inadequate diagnostic accuracy for pleural fluid MTB culture
    K. Inadequate diagnostic accuracy for pleural biopsy
    L. Inadequate diagnostic accuracy for pleural fluid biomarkers (e.g. ADA)
    M. Others, please specify
