# Supplementary material for: A survey of respiratory physicians on investigating and managing tuberculous pleuritis
Source: Sci Rep. 2026 Jun 10;16:26533. doi: 10.1038/s41598-026-57407-3 (PMC13503735; doi:10.1038/s41598-026-57407-3)
Supplement: Supplementary file 2 [file 41598_2026_57407_MOESM1_ESM.docx]

Supplementary Table 1. Definition of TB burden and the reported TB burden in the region of practice of respondents.

| **Category** | **Definition** | **Regions** | **Incidence in year 2024 (per 100,000 population) [1,2]** |
| --- | --- | --- | --- |
| High | Incidence rate of more than 100 cases per 100,000 population per year | Philippines  Myanmar  Indonesia  India  Vietnam  Thailand | 624.9  481.8  382.2  186.6  182.5  145.7 |
| Intermediate | Incidence rate of 10-100 cases per 100,000 population per year | Malaysia  Hong Kong  Macau  Mainland China  Singapore  South Korea  Taiwan  Türkiye | 96.0  66  59  49.1  43.3  34.7  28  13.1 |
| Low | Incidence rate of fewer than 10 cases per 100,000 population per year | Australia | 6.9 |

Data for Macau, Hong Kong and Taiwan were obtained from the World Bank [2], with only integer values available.

*TB: tuberculosis*

Supplementary Table 2. Correlation between reported and perceived TB burden of the region of practice among the respondents.

|  | | **Perceived TB burden** | | |
| --- | --- | --- | --- | --- |
|  |  | **Low** | **Intermediate** | **High** |
| Reported TB burden | Low, n (%) | 5 (1.2) | 0 (0.0) | 0 (0.0) |
|  | Intermediate, n (%) | 88 (21.3) | 150 (36.2) | 67 (16.2) |
|  | High, n (%) | 1 (0.2) | 11 (2.7) | 92 (22.2) |

*TB: tuberculosis*

Supplementary Table 3. Univariable analysis of factors that correlate with the consideration of TBP as a differential diagnosis.

| **Variables** | **Odds ratio** | **95% CI** | **p-value** |
| --- | --- | --- | --- |
| Male  Female | 0.82  (Reference) | 0.50 – 1.34 | 0.422 |
| Age, years  ≤ 29  30 – 39  40 – 49  50 – 59  60 – 69  ≥ 70 | 1.51  0.99  0.82  1.09  0.48  (Reference) | 0.57 – 4.00  0.60 – 1.62  0.49 – 1.38  0.55 – 2.15  0.04 – 5.40 | 0.413  0.965  0.461  0.807  0.555 |
| Experience of practice in respiratory medicine, years ^1^  ≤ 5  6 – 10  11 – 15  16 – 19  ≥ 20 | (Reference)  0.90  1.00  1.07  1.66 | 0.52 – 1.58  0.55 – 1.82  0.48 – 2.41  0.81 – 3.39 | 0.721  0.995  0.867  0.167 |
| Major site of practice  Private institute (non-teaching hospital)  Public institute (non-teaching hospital)  University-affiliated or teaching hospital  Others ^2^ | 0.97  0.76  1.25  (Reference) | 0.43 – 2.19  0.46 – 1.26  0.77 – 2.03 | 0.942  0.292  0.372 |
| Frequency of encountering patients with new-onset unilateral pleural effusion  Never  < 5 / year  5 – 10 / year  1 – 4 / month  5 – 10 / month  > 10 / month | (Reference)  0.97  2.69  1.19  0.69  0.74 | 0.35 – 2.67  1.12 – 6.48  0.71 – 2.00  0.39 – 1.20  0.42 – 1.31 | 0.955  0.027  0.506  0.189  0.303 |
| Proportion of TBP among patients with new-onset unilateral pleural effusion  < 5%  5 – 10%  11 – 25%  26 – 50%  > 50% | (Reference)  0.53  1.51  1.50  5.70 | 0.31 – 0.92  0.81 – 2.84  0.84 – 2.70  1.35 – 24.07 | 0.024  0.196  0.173  0.018 |
| Reported TB burden  High  Intermediate  Low | 2.62  0.43  (Reference) | 0.41 – 1.19  0.98 – 2.77 | 0.185  0.062 |
| Perceived TB burden  High  Intermediate  Low | 1.08  0.85  (Reference) | 0.65 – 1.78  0.52 – 1.40 | 0.778  0.525 |

*CI: confidence interval; TB: tuberculosis; TBP: tuberculous pleuritis*

^1^ Includes the training period

^2^ Other sites of practice include all of the above (1), private and teaching hospitals (1) and public and teaching hospitals (1)

Supplementary Table 4. Pleural investigations that are commonly ordered during the first diagnostic thoracentesis for new-onset unilateral pleural effusion. ^1^

| Pleural investigations | Respondents answering “most of the time” or “always”  (n=414) | Respondents answering “not available”  (n=414) | Reported high TB burden ^2^ | Reported low / intermediate TB burden ^2^ | p-value ^3^ | Respondents “most of the time” or “always” consider TBP as a differential diagnosis ^2^ | Respondents “never”, “sometimes” or “about half of the time” consider TBP as a differential diagnosis ^2^ | p-value ^4^ |
| --- | --- | --- | --- | --- | --- | --- | --- | --- |
| Red cell count, n (%) | 285 (68.8) | 19 (4.6) | 56 / 102 (54.9) | 229 / 293 (78.2) | < 0.001 | 238 / 319 (74.6) | 47 / 76 (61.8) | 0.026 |
| White blood / nucleated cell total cell count, n (%) | 377 (91.1) | 9 (2.2) | 97 / 104 (93.3) | 280 / 301 (93.0) | 0.932 | 311 / 326 (95.4) | 66 / 79 (83.5) | < 0.001 |
| White blood / nucleated cell differential count, n (%) | 363 (87.7) | 17 (4.1) | 96 / 104 (92.3) | 267 / 293 (91.1) | 0.711 | 299 / 320 (93.4) | 64 / 77 (83.1) | 0.004 |
| Light’s criteria (total protein and LDH), n (%) | 393 (94.9) | 4 (1.0) | 97 / 103 (94.2) | 296 / 307 (96.4) | 0.323 | 322 / 330 (97.6) | 71 / 80 (88.8) | < 0.001 |
| Albumin, n (%) | 292 (70.5) | 12 (2.9) | 57 / 101 (56.4) | 235 / 301 (78.1) | < 0.001 | 249 / 323 (77.1) | 43 / 79 (54.4) | < 0.001 |
| ADA, n (%) | 338 (81.6) | 17 (4.1) | 62 / 95  (65.3) | 276 / 302 (91.4) | < 0.001 | 287 / 318 (90.3) | 51 / 79 (64.6) | < 0.001 |
| pH, n (%) | 233 (56.3) | 49 (11.8) | 31 / 88  (35.2) | 202 / 277 (72.9) | < 0.001 | 193 / 289 (66.8) | 40 / 76 (52.6) | 0.022 |
| Glucose, n (%) | 346 (83.6) | 3 (0.7) | 75 / 104 (72.1) | 271 / 307 (88.3) | < 0.001 | 290 / 330 (87.9) | 56 / 81 (69.1) | < 0.001 |
| Bacterial culture (in plain bottle), n (%) | 327 (79.0) | 4 (1.0) | 68 / 103 (66.0) | 259 / 307 (84.4) | < 0.001 | 275 / 331 (83.1) | 52 / 79 (65.8) | < 0.001 |
| Bacterial culture (in culture bottle), n (%) | 199 (48.1) | 11 (2.7) | 42 / 99  (42.4) | 157 / 304 (51.6) | 0.111 | 170 / 325 (52.3) | 29 / 78 (37.2) | 0.016 |
| AFB smear, n (%) | 306 (73.9) | 5 (1.2) | 53 / 104 (51.0) | 253 / 305 (83.0) | < 0.001 | 249 / 328 (75.9) | 57 / 81 (70.4) | 0.303 |
| MTB culture, n (%) | 239 (57.7) | 33 (8.0) | 44 / 99  (44.4) | 195 / 282 (69.1) | < 0.001 | 187 / 301 (62.1) | 52 / 80 (65.0) | 0.637 |
| MTB PCR, including GeneXpert or Xpert Ultra, n (%) | 186 (44.9) | 22 (5.3) | 57 / 103 (55.3) | 129 / 289 (44.6) | 0.062 | 163 / 313 (52.1) | 23 / 79 (29.1) | < 0.001 |
| Cytology, paraffin embedded pathological analysis, n (%) | 358 (86.5) | 4 (1.0) | 87 / 102 (85.3) | 271 / 308 (88.0) | 0.479 | 301 / 331 (90.9) | 57 / 79 (72.2) | < 0.001 |
| Pleural biopsy for histology, n (%) | 110 (26.6) | 10 (2.4) | 20 / 100 (20.0) | 90 / 304 (29.6) | 0.061 | 101 / 325 (31.1) | 9 / 79 (11.4) | < 0.001 |
| Pleural biopsy for MTB culture, n (%) | 158 (38.2) | 26 (6.3) | 41 / 100 (41.0) | 117 / 288 (40.6) | 0.948 | 129 / 310 (41.6) | 29 / 78 (37.2) | 0.476 |
| Sputum for AFB smear and/or MTB culture, n (%) | 315 (76.1) | 9 (2.2) | 70 / 104 (67.3) | 245 / 301 (81.4) | 0.003 | 269 / 325 (82.8) | 46 / 80 (57.5) | < 0.001 |

^1^ Row and column percentages may not sum to exactly 100% due to rounding.

^2^ Percentages are calculated with respondents reporting that corresponding tests are available as the denominator.

^3^ Comparison between reported high and low / intermediate TB burden for respondents’ regions of practice.

^4^ Comparison between respondents who “most of the time” or “always” consider TBP as a differential diagnosis and other respondents.

*ADA: adenosine deaminase; AFB: acid-fast bacilli; LDH: lactate dehydrogenase; MTB: Mycobacterium tuberculosis; PCR: polymerase chain reaction*

Supplementary Table 5. Unavailability of TBP-related pleural investigations according to the region of practice.

| **Regions** | **Number of respondents** | **TBP-related pleural investigations that are not available** | | | | | |
| --- | --- | --- | --- | --- | --- | --- | --- |
|  |  | **Pleural fluid AFB** | **Pleural fluid MTB culture** | **Pleural fluid MTB PCR** | **Pleural fluid ADA** | **Pleural tissue MTB culture** | **Pleural biopsy service** |
| Mainland China, n (%) | 166 (40.1) | 4 (2.4) | 28 (16.9) | 21 (12.7) | 3 (1.8) | 21 (12.7) | 20 (12.0) |
| Hong Kong, n (%) | 43 (10.4) | 0 (0.0) | 0 (0.0) | 0 (0.0) | 0 (0.0) | 0 (0.0) | 0 (0.0) |
| Taiwan, n (%) | 33 (8.0) | 0 (0.0) | 0 (0.0) | 0 (0.0) | 3 (9.1) | 1 (3.0) | 0 (0.0) |
| Thailand, n (%) | 33 (8.0) | 0 (0.0) | 0 (0.0) | 0 (0.0) | 1 (3.0) | 0 (0.0) | 0 (0.0) |
| India, n (%) | 24 (5.8) | 0 (0.0) | 0 (0.0) | 0 (0.0) | 1 (4.2) | 0 (0.0) | 0 (0.0) |
| Philippines, n (%) | 24 (5.8) | 0 (0.0) | 4 (16.7) | 0 (0.0) | 5 (20.8) | 3 (12.5) | 2 (8.3) |
| Malaysia, n (%) | 22 (5.3) | 0 (0.0) | 0 (0.0) | 0 (0.0) | 1 (4.5) | 0 (0.0) | 0 (0.0) |
| South Korea, n (%) | 21 (5.1) | 1 (4.8) | 0 (0.0) | 0 (0.0) | 0 (0.0) | 0 (0.0) | 0 (0.0) |
| Indonesia, n (%) | 21 (5.1) | 0 (0.0) | 1 (4.8) | 1 (4.8) | 1 (4.8) | 1 (4.8) | 6 (28.6) |
| Singapore, n (%) | 15 (3.6) | 0 (0.0) | 0 (0.0) | 0 (0.0) | 0 (0.0) | 0 (0.0) | 0 (0.0) |
| Australia, n (%) | 5 (1.2) | 0 (0.0) | 0 (0.0) | 0 (0.0) | 1 (20.0) | 0 (0.0) | 0 (0.0) |
| Macau, n (%) | 4 (1.0) | 0 (0.0) | 0 (0.0) | 0 (0.0) | 0 (0.0) | 0 (0.0) | 0 (0.0) |

*ADA: adenosine deaminase; MTB: Mycobacterium tuberculosis; PCR: polymerase chain reaction; TB: tuberculosis; TBP: tuberculous pleuritis*

Supplementary Table 6. Correlation between the request TBP-related investigations and the diagnosis pattern of TBP.

| **TBP-related investigations that are “most of the time” or “always” ordered** | **Test available** | **Respondents preferred the diagnosis of definite TBP** | **Respondents allowed the diagnosis of probable TBP or made clinically** | **p-value** |
| --- | --- | --- | --- | --- |
| Pleural fluid AFB stain, n (%) | 409 (97.3) | 197 (78.8) | 109 (68.6) | 0.020 |
| Pleural fluid MTB culture, n (%) | 381 (92.0) | 152 (65.5) | 87 (58.4) | 0.160 |
| Pleural fluid MTB PCR, n (%) | 392 (94.7) | 113 (47.3) | 73 (47.7) | 0.933 |
| Pleural fluid ADA, n (%) | 397 (95.9) | 212 (87.6) | 126 (81.3) | 0.085 |
| Pleural tissue for histology, n (%) | 404 (97.6) | 89 (35.9) | 21 (13.5) | < 0.001 |
| Pleural tissue for MTB culture, n (%) | 388 (93.7) | 99 (41.8) | 59 (39.1) | 0.598 |
| Sputum for AFB stain and/or MTB culture, n (%) | 405 (97.8) | 196 (79.4) | 119 (75.3) | 0.341 |

*ADA: adenosine deaminase; AFB: acid-fast bacilli; MTB: Mycobacterium tuberculosis; TBP: tuberculous pleuritis; PCR: polymerase chain reaction*

Supplementary Table 7. Perception of the diagnostic accuracy of different tests for diagnosing TBP according to the reported TB prevalence of respondents.

| Parameters | All respondents  (n = 414) | Respondents from high TB burden regions  (n = 104) | Respondents from low-to-intermediate TB burden regions  (n =310) | p-value ^1^ |
| --- | --- | --- | --- | --- |
| PT histology, n (%)  > 75%  50 – 74%  25 – 49%  10 – 24%  < 10% | 200 (48.3)  100 (24.2)  69 (16.7)  29 (7.0)  16 (3.9) | 48 (46.2)  26 (25.0)  20 (19.2)  7 (6.7)  3 (2.9) | 152 (49.0)  74 (23.9)  49 (15.8)  22 (7.1)  13 (4.2) | 0.897 |
| PT MTB culture, n (%)  > 75%  50 – 74%  25 – 49%  10 – 24%  < 10% | 108 (26.1)  68 (16.4)  92 (22.2)  66 (15.9)  80 (19.3) | 30 (28.8)  18 (17.3)  21 (20.2)  19 (18.3)  16 (15.4) | 78 (25.2)  50 (16.1)  71 (22.9)  47 (15.2)  64 (20.6) | 0.678 |
| PF MTB PCR, n (%)  > 75%  50 – 74%  25 – 49%  10 – 24%  < 10% | 69 (16.7)  68 (16.4)  96 (23.2)  99 (23.9)  82 (19.8) | 17 (16.3)  18 (17.3)  26 (25.0)  30 (28.8)  13 (12.5) | 52 (16.8)  50 (16.1)  70 (22.6)  69 (22.3)  69 (22.3) | 0.246 |
| PF MTB culture, n (%)  > 75%  50 – 74%  25 – 49%  10 – 24%  < 10% | 61 (14.7)  38 (9.2)  73 (17.6)  115 (27.8)  127 (30.7) | 14 (13.5)  10 (9.6)  19 (18.3)  32 (30.8)  29 (27.9) | 47 (15.2)  28 (9.0)  54 (17.4)  83 (26.8)  98 (31.6) | 0.908 |
| PF AFB, n (%)  > 75%  50 – 74%  25 – 49%  10 – 24%  < 10% | 28 (6.8)  19 (4.6)  46 (11.1)  75 (18.1)  246 (59.4) | 4 (3.8)  5 (4.8)  7 (6.7)  22 (21.2)  66 (63.5) | 24 (7.7)  14 (4.5)  39 (12.6)  53 (17.1)  180 (58.1) | 0.263 |

^1^ Comparison between respondents from high TB burden regions and those from low-to-intermediate TB burden regions.

*AFB: acid-fast bacilli; MTB: Mycobacterium tuberculosis; PCR: polymerase chain reaction; PF: pleural fluid; PT: pleural tissue*

Supplementary Table 8. Utilization of pleural fluid biomarkers for diagnosing TBP.

| **Questions** | **Respondents** |
| --- | --- |
| Diseases that can potentially elevate the pleural fluid ADA levels, n (%)  TBP  Pleural infections (CPPE / empyema)  MPE due to solid cancers  Heart failure  Lymphoma  None of the above | 405 (97.8)  317 (76.6)  205 (49.5)  22 (5.3)  219 (52.9)  1 (0.2) |
| Cutoff levels of pleural fluid ADA, U/L  Upper limit  Lower limit | 66.3 ± 27.1  38.2 ± 14.8 |
| Types of pleural fluid biomarkers used to diagnose TBP other than ADA, n (%)  Nil others  IFN-gamma  TNF-alpha  IGRA  Interleukins (e.g. IL-2, IL-27)  LDH / ADA ratio | 357 (86.2)  15 (3.6)  3 (0.7)  13 (3.1)  2 (0.5)  26 (6.3) |
| Pleural fluid biomarkers with greatest confidence in diagnosis of TBP *(optional question)*, n (%)  ADA  IFN-gamma  TNF-alpha  IGRA  Interleukins (e.g. IL-2, IL-27)  LDH / ADA ratio | 46 (63.0)  2 (2.7)  0 (0.0)  9 (12.3)  1 (1.4)  9 (12.3) |

*ADA: adenosine deaminase; AFB: acid-fast bacilli; CPPE: complicated parapneumonic effusion; IFN-gamma: interferon-gamma; IGRA: interferon-gamma release assay; IL: interleukin; LDH: lactate dehydrogenase; TBP: tuberculous pleuritis; TNF-alpha; tumour necrosis factor-alpha*

Supplementary Table 9. Factors that may affect the decision of empirical anti-TB treatment in patients with probable TBP.

| **Factors** | **Respondents answering “most of the time” or “always”**  **(n=414)** | **Reported high TB burden**  **(n=104)** | **Reported low to intermediate TB burden**  **(n=310)** | **p-value ^1^** | **Respondents “most of the time” or “always” consider TBP as a differential diagnosis**  **(n=333)** | **Respondents “never”, “sometimes” or “about half of the time” consider TBP as a differential diagnosis**  **(n=81)** | **p-value ^2^** |
| --- | --- | --- | --- | --- | --- | --- | --- |
| Old age (> 65 years old), n (%) | 155 (37.4) | 44 (42.3) | 111 (35.8) | 0.236 | 118 (35.4) | 37 (45.7) | 0.088 |
| Poor performance status, n (%) | 128 (30.9) | 40 (38.5) | 88 (28.4) | 0.054 | 97 (29.1) | 31 (38.3) | 0.110 |
| Absence of compatible symptoms, e.g. fever, pleurisy, n (%) | 128 (30.9) | 24 (23.1) | 104 (33.5) | 0.045 | 106 (31.8) | 22 (27.2) | 0.415 |
| Presence of chronic liver disease or abnormal LFT, n (%) | 101 (24.4) | 19 (18.3) | 82 (26.5) | 0.093 | 83 (24.9) | 18 (22.2) | 0.611 |
| Presence of chronic renal disease or abnormal RFT, n (%) | 132 (31.9) | 37 (35.6) | 95 (30.6) | 0.350 | 107 (32.1) | 25 (30.9) | 0.826 |
| Concern of polypharmacy and risk of drug-drug interaction, n (%) | 114 (27.5) | 31 (29.8) | 83 (26.8) | 0.549 | 95 (28.5) | 19 (23.5) | 0.359 |
| Lack of radiographic changes suggestive of pulmonary TB in the lungs by chest images, n (%) ^3^ | 145 (35.0) | 43 (41.3) | 102 (32.9) | 0.118 | 122 (36.6) | 23 (28.4) | 0.163 |
| Inadequate confidence in excluding other alternative explanations for raised ADA, n (%) | 70 (16.9) | 16 (15.4) | 54 (17.4) | 0.632 | 61 (18.3) | 9 (11.1) | 0.121 |
| Lack of or waiting for confirmatory test to diagnose TBP, n (%) ^4^ | 109 (26.3) | 36 (34.6) | 73 (23.5) | 0.027 | 84 (25.2) | 25 (30.9) | 0.301 |

^1^ Comparison between reported high and low to intermediate TB burden for respondents’ regions of practice.

^2^ Comparison between respondents who “most of the time” or “always” consider TBP as a differential diagnosis and other respondents.

^3^ Radiographic changes of pulmonary TB, including consolidation, fibronodular infiltrates or cavitary lesions.

^4^ Confirmatory test results, including positive MTB culture or PCR in pleural fluid or pleural tissue, or compatible histological findings.

*ADA: adenosine deaminase; LFT: liver function test; MTB: Mycobacterium tuberculosis; PCR: polymerase chain reaction; RFT: renal function test; TB: tuberculosis; TBP: tuberculous pleuritis*

Supplementary Table 10. Management following the initial diagnostic thoracentesis or when TBP is confirmed.

| **Questions** | **Respondents** |
| --- | --- |
| “If a pleural biopsy is needed in this patient, what is your usual practice?”, n (%)  Non-ultrasound guided pleural biopsy by respiratory physicians  Real-time ultrasound-guided pleural biopsy by respiratory physicians  Real-time image-guided (CT or ultrasound) pleural biopsy by radiologists  Pleuroscopy (medical thoracoscopy) guided pleural biopsy by respiratory physicians  Surgical pleural biopsy by thoracic surgeons  Pleural biopsy is not available in my institute | 44 (10.6)  80 (19.3)  40 (9.7)  205 (49.5)  30 (7.2)  15 (3.6) |
| “From your experience, how many of your patients required a revision of diagnosis for pleural effusion due to response failure to empirical anti-TB treatment?”, n (%)  None  1 – 9%  10 – 24%  25 – 49%  50 – 74%  >= 75% | 92 (22.2)  166 (40.1)  98 (23.7)  32 (7.7)  8 (1.9)  18 (4.3) |
| “If there is residual pleural effusion after diagnosing TBP, would you consider drainage of the effusion in addition to anti-TB treatment?”, n (%)  No  Effusion size < 25%  Effusion size > 25%  Effusion size > 50%  Symptom-based, regardless of the effusion size | 57 (13.8)  72 (17.4)  105 (25.4)  72 (17.4)  108 (26.1) |

*CT: computed tomography; TB: tuberculosis; TBP: tuberculous pleuritis*

Supplementary Table 11. Service efficiency of TBP-specific pleural investigations.

| **Parameters** | **Respondents** | **Median waiting time, working days (IQR)** |
| --- | --- | --- |
| Average turnaround time for pleural fluid ADA in the respondents’ institute, n (%)  Within 2 working days  3 – 5 working days  6 – 10 working days  > 10 working days  Test not available | 230 (55.6)  94 (22.7)  48 (11.6)  20 (4.8)  22 (5.3) | N/A |
| Average turnaround time for pleural tissue histology in the respondents’ institute, n (%)  Within 2 working days  3 – 5 working days  6 – 10 working days  > 10 working days  Test not available | 21 (5.1)  200 (48.3)  140 (33.8)  41 (9.9)  12 (2.9) | N/A |
| Availability of biopsy services in respondents’ institute, n (%)  Non-ultrasound guided pleural biopsy by respiratory physicians  Real-time ultrasound-guided pleural biopsy by respiratory physicians  Real-time image-guided (CT or ultrasound) pleural biopsy by radiologists  Pleuroscopy (medical thoracoscopy) guided pleural biopsy by respiratory physicians  Surgical pleural biopsy by thoracic surgeons  None of the above | 178 (43.0)  254 (61.4)  240 (58.0)  287 (69.3)  205 (49.5)  28 (6.8) | 1.0 (1.0 – 2.0)  1.0 (1.0 – 3.0)  1.0 (1.0 – 7.0)  3.0 (2.0 – 7.0)  5.0 (3.0 – 10.0)  N/A |

*ADA: adenosine deaminase; CT: computed tomography; IQR: interquartile range; N/A: not applicable*

References:

1. <https://worldhealthorg.shinyapps.io/tb_profiles/> (assessed on 3 Feb 2026)
2. <https://data.worldbank.org/indicator/SH.TBS.INCD> (assessed on 3 Feb 2026)
